# Lived Experiences of Patients With Rare Diseases and Healthcare System Barriers: A Phenomenological Study

**DOI:** 10.1155/jonm/6754802

**Published:** 2026-06-29

**Authors:** Pedro Soriano-Martin, Cristofer Ruiz-González, Antonio Javier Alias-Castillo, Pablo Roman, Adrian Martinez-Ortigosa, Daniel Bertini-Pérez, Isabel Font Jímenez

**Affiliations:** ^1^ Department of Nursing, Faculty of Medicine, Health and Sports, Universidad Europea de Madrid, Villaviciosa de Odón, Spain, uem.es; ^2^ Torrecárdenas University Hospital, Almeria, Andalusia, 04009, Spain, hospitaltorrecardenas.es; ^3^ Advances and Innovation in Health Research Group (AIS, CTS-1114), Almeria, Andalusia, Spain, ual.es; ^4^ Department of Nursing Science, Physiotherapy and Medicine, Faculty of Health Sciences, University of Almeria, Almeria, Andalusia, Spain, ual.es; ^5^ Department of Nursing, Faculty of Nursing and Podiatry, University of Valencia, Valencia, Spain, uv.es; ^6^ CARE360 Research Group, University of Almeria, Andalusia, Almeria, Spain, ual.es

**Keywords:** disease management, health information management, healthcare disparities, rare diseases

## Abstract

**Aim:**

To explore the lived experiences and perceptions of patients with rare diseases (RD) in relation to the disease process and its management by the healthcare system.

**Background:**

Although each RD individually affects fewer than 0.05% of the population, collectively RD affect between 3.5% and 5.9% of the global population, representing approximately 400 million people worldwide. Most RD are chronic, progressive, and debilitating, with 80% having a genetic origin. Despite advances, diagnosing RD remains complex, often taking 4 to 8 years, worsening patient outcomes and increasing healthcare costs. Furthermore, 95% of RD lack approved treatments, presenting significant challenges for both patients and healthcare systems.

**Methods:**

An interpretative phenomenological qualitative study following Gadamer’s hermeneutic framework was conducted. Semistructured, in‐depth interviews were conducted between February 2022 and January 2024. Seventeen patients with RD were recruited using purposeful sampling. ATLAS.ti v.9 software was used solely to organize and manage the data during the analysis process.

**Results:**

Two main interpretive themes emerged: (1) RD: a desperate struggle against abstraction and hindrance, describing the emotional burden, diagnostic delays, and social consequences faced by patients with RD and (2) management and handling of RD by the healthcare system, highlighting professional unpreparedness, lack of coordination, and the key role patients and caregivers play in guiding care and sharing knowledge, alongside the emergence of peer support, digital tools, and social media as facilitators.

**Conclusion:**

This study highlights the significant barriers patients with RD face, from diagnosis to treatment. Healthcare systems struggle with insufficient knowledge and resources, hindering effective care. It is essential for professionals to acquire specialized skills and for resource allocation to improve in order to address RD as a public health concern.

**Implications for Nursing Management:**

As part of an interprofessional team, nursing professionals play a vital role in supporting patients with RD throughout the diagnostic journey, treatment, and management. This study highlights the need for nurses to address not only clinical but also psychosocial and informational challenges, including guiding patients in the safe and effective use of social media as a source of support, information, and empowerment.

**Patient and Public Contribution:**

Patients contributed as participants in the study by sharing their lived experiences through in‐depth interviews. No patients or members of the public were involved in the design, conduct, reporting, or dissemination plans of this research.

## 1. Background

Rare diseases (RD) constitute a subject of great scientific and healthcare interest that has been increasing in recent years [1, 2]. They are defined as a group of pathologies whose main characteristic is their low prevalence in the population, which is usually less than 0.05% [3]. Most are chronic, progressive, and debilitating diseases and can lead to significant morbidity and mortality [4]. RD are uncommon among individuals, but their global prevalence is estimated to be between 3.5% and 5.9% [3, 5], affecting approximately 400 million people [6]. In Europe, there are approximately 30 million individuals suffering from an RD, with around 920,000 patients located in Spain [7]. However, prevalence data are not consistent, both due to the lack of consensus in establishing a suitable definition and the difficulties in detecting and recording these pathologies [8]. To date, between 6000 and 8000 RD have been identified worldwide, and their number is increasing, with around 250 new diseases being classified as rare each year [2, 9].

In terms of etiology, approximately 80% of RD have a genetic cause, and less frequently, they result from infectious, immunological, degenerative, or proliferative diseases [10]. This group of pathologies can appear at any stage of the patient’s life, with the vast majority manifesting during childhood, although sometimes the diagnosis is established in adolescence or adulthood [1, 3]. Despite increasing knowledge, resources, and research advances [11, 12], the diagnosis of these diseases remains complex [13]. Thus, it is estimated that the average time to establish the diagnosis of an RD ranges from 4 to 8 years, leading to a negative impact on both patients and their families, a high cost for the healthcare system, and a potential worsening of the disease prognosis [13–15]. The clinical manifestations associated with RD are characterized by their considerable variability within the same condition and among individuals with the same pathology, with multiple impairments in patients’ abilities and the presence of weakness and disability [16, 17]. In addition, there is a significant deficit in measures and therapeutic options for RD, with approximately 95% of them lacking approved specific treatments [18].

The difficulty in establishing a precise diagnosis, the chronic nature of expensive treatments, and recurrent hospital admissions entail a significant impact on both the patient and the healthcare system [19]. In fact, a recent study demonstrates that the economic burden of RD is approximately tenfold higher than that of common diseases [20, 21]. Despite the challenging obstacles faced by the population with RD, the marginalization of this sector in the healthcare system and in social and healthcare policies continues to be a clear reality in contemporary society [22, 23].

Different studies have tried to understand the perspective of patients with RD on their health status and the course of their illness [24–33], as well as the perceptions and experiences of health professionals in contact with RD [34–38]. However, much of this literature is disease‐specific or focuses primarily on biomedical outcomes. Cross‐cutting qualitative analyses that center the lived experience across different RD remain scarce. Moreover, there is limited examination of how people with RD engage with health services, experience structural barriers, and make sense of their illness beyond clinical parameters. Our study seeks to fill this gap by capturing a broader, patient‐centered understanding of what it means to live with an RD and to interact with healthcare systems that are often ill‐equipped to meet their needs.

In this context, nurses are increasingly positioned as key professionals in the care and support of patients with RD, given their proximity to the patient and their role in care continuity. Understanding the specific barriers that people with RD face within the healthcare system is essential for nurses: it enables them to anticipate care gaps, advocate effectively on behalf of patients, and tailor their practice to the distinctive needs of this population. Their competencies encompass case management, disease‐specific knowledge, health education, a humanized approach, and digital proficiency [39]. These evolving responsibilities demand advanced training and greater autonomy in clinical decision‐making [40], enabling equitable and high‐quality care at all levels of the system. Understanding patients’ lived experiences is thus essential to inform the development of nursing roles and ensure that care strategies respond effectively to the complex realities faced by those with RD. Accordingly, this study was guided by the following research question: How do patients with RD perceive and experience their disease process and its management by the healthcare system?

## 2. Methods

### 2.1. Aim

The aim of this study was to explore the lived experiences and perceptions of patients with RD in relation to the disease process and its management by the healthcare system.

### 2.2. Design

Between February 2022 and January 2024, a qualitative study was conducted following a hermeneutic phenomenological approach. The research question guiding the study was: How do patients with RD perceive and experience their disease process and the management by health services? The study was designed on the assumption that RD requires a comprehensive and in‐depth exploration focused on patients′ lived experiences, as this perspective is essential to fully understand the complexity of these conditions.

To achieve this, Gadamer’s hermeneutic framework was used, which emphasizes that human experiences and phenomena are inseparable from language and must be interpreted through dialog with others, allowing different perspectives to merge. Through such dialog and the understanding of individual narratives, meaning emerges [41, 42]. The study followed the methodological adaptation of Gadamer’s philosophy proposed by Valerie Fleming [43]. First, the phenomenon under investigation, the experience of living with an RD, was firmly rooted in the lifeworld of the participants, making it appropriate for phenomenological inquiry. Second, the research team engaged in a process of reflexivity, identifying and acknowledging their own pre‐understandings related to RD, healthcare access, and patient vulnerability. As Gadamer asserts, these pre‐understandings are not obstacles to knowledge, but rather integral to the interpretive process. Finally, the team’s professional background, encompassing clinical care, qualitative research, and patient advocacy, enhanced both the richness of data collection and the depth of interpretation.

The study followed the Standards for Reporting Qualitative Research (SRQR) [44].

### 2.3. Participants and Settings

Participants were recruited through two pathways: (i) Spanish RD patient associations and (ii) a specialized RD reference unit at a public university hospital in Madrid. Initial contact was established through the coordinators of the participating associations and the reference clinical unit. No prior relationship existed between the researchers and any participant before recruitment. All eligible individuals who were approached agreed to participate; no potential participant refused. The study participants included patients with various types of RD (Table 1). A purposeful sampling method was applied, with the following inclusion criteria: (i) a confirmed diagnosis of an RD and (ii) the provision of signed informed consent to participate in the study. No exclusion criteria were defined.

**TABLE 1:** Sociodemographic characteristics of participants.

Code	Age	Sex	Disease	Months from symptom onset to diagnosis
P1	41	M	Glycogenosis (glycogen storage disease)	336
P2	29	F	Cystic fibrosis	2
P3	35	F	Mitochondrial disease	168
P4	32	M	Eosinophilic esophagitis	48
P5	50	F	Ehlers–Danlos syndrome	60
P6	28	M	Wolfram syndrome	60
P7	40	F	Complex regional pain syndrome	48
P8	34	F	Peripartum cardiomyopathy	1
P9	42	M	Congenital multiple arthrogryposis	0
P10	41	F	Idiopathic intracranial hypertension + Tarlov cysts	168
P11	36	M	Gaucher disease	120
P12	30	F	Amyotrophic lateral sclerosis	30
P13	45	M	Fanconi anemia	240
P14	44	F	Pompe disease	96
P15	50	M	Huntington’s disease	180
P16	27	F	Wilson’s disease	60
P17	33	M	Marfan syndrome	120

*Note:* F: female; M: male.

### 2.4. Data Collection

Data collection took place between February 2022 and January 2024 through semistructured in‐depth interviews. The interview guide was collaboratively developed by the research team, based on prior literature and the study objectives (Supporting File 1). Seventeen in‐depth interviews were conducted face‐to‐face in a private room within the collaborating clinical unit or, when preferred by the participant, at a quiet location designated by them (e.g., a meeting room at the patient association premises), and led by two researchers, one of whom was an expert in conducting the dialog, and an observer who collaborated and took field notes. The interviews were audio‐recorded, and the average length of the interviews was 65 min. Data collection continued until data saturation was reached after fifteen interviews, with two additional interviews conducted to confirm saturation, as no new meaningful information emerged. Prior to analysis, participants had the opportunity to review the transcripts.

### 2.5. Ethical Considerations

Approval for the study was granted by the Ethics Committee of the Universidad Europea de Madrid (CIPI/22.009), and all ethical considerations outlined in the Declaration of Helsinki were adhered to. Before obtaining consent, participants were informed about the study’s nature, their voluntary involvement, and their right to withdraw at any time without providing a reason. The rights to privacy, confidentiality, and anonymity were protected through the use of alphanumeric identifiers assigned sequentially to each participant (PX), and transcripts were reviewed to ensure no indirect identifiers were included. Audio recordings and transcripts were stored on an encrypted, password‐protected institutional server, with access restricted to the research team, until dissemination of the study results, after which all files will be permanently destroyed in accordance with institutional policy.

### 2.6. Data Analysis

Data were analyzed using Gadamer’s hermeneutic philosophy, following the five‐phase structure proposed by Fleming and colleagues [43]: (i) deciding on a question; (ii) identifying pre‐understandings; (iii) obtaining understanding through dialog with the participants; (iv) obtaining understanding through dialog with the text; and (v) establishing reliability.

First, researchers clarified their pre‐understandings through reflexive journaling and discussion prior to analysis in order to minimize potential bias arising from previous clinical experience. Transcripts were read and re‐read manually to gain a naïve understanding while listening to audio recordings and consulting field notes to remain close to the participants’ voices. Each interview was analyzed independently to respect the uniqueness of the lived experience. In a second phase, meaningful units were inductively identified and interpreted in dialog with the whole text, allowing for a deeper, contextualized understanding. Through iterative discussion and comparison among researchers, shared interpretations were developed—illustrating the fusion of horizons between participants and the research team. ATLAS.ti 9 was used solely as a tool for organizing data (coding and grouping), but interpretation emerged from hermeneutic reflection and dialog within the research team rather than from software output. Following data analysis, a summary of the emerging interpretive themes was shared with participants, who were invited to confirm whether the interpretations resonated with their experiences.

### 2.7. Rigor

Methodological rigor was ensured using Lincoln and Guba’s criteria: credibility, transferability, dependability, and confirmability [45]. Credibility was supported through triangulation of researchers, which included independent coding, team discussions to resolve discrepancies, and review by a third researcher. Participant validation was conducted by offering participants the opportunity to review and confirm their transcripts. To enhance transferability, specific selection criteria and comprehensive demographic information were provided. To enhance the phenomenological representation of participants’ experiences, pseudonyms were assigned that reflect contextual or personal characteristics, adding narrative depth while preserving anonymity. Dependability was supported through detailed documentation of analytical decisions and maintenance of an audit trail, and confirmability was ensured by reflexive journaling and external review of the interpretive coding and meaning‐development process.

## 3. Findings

A total of 17 individuals diagnosed with an RD participated in the study, with a mean age of 37.47 years (range 27–50) and a mean time from symptom onset to diagnosis of 89.51 months (range 0–336). Participants showed a broad diversity of RD diagnoses, reflecting the clinical heterogeneity that characterizes RD and providing a rich basis for phenomenological interpretation. The analysis of the data revealed two main interpretative themes that characterize patients’ perceptions and experiences of RD and their management by the healthcare system.

### 3.1. Theme 1. RD: A Desperate Struggle Against Abstraction and Hindrance

Patients with RD are immersed in a situation of ignorance and frustration until they receive the diagnostic label of their pathology. Throughout this process, the feeling of impersonality and the lack of a clear explanation for their health status further exacerbate the perceived deterioration. In addition, multiple barriers will hinder the optimal evolution of the pathology and delay the complex management required. Thus, the significant challenge of suffering from a rare and debilitating disease becomes just one more of the obstacles for the patient to deal with in their daily life.

#### 3.1.1. Subtheme 1.1. Experiencing Diagnostic Uncertainty

When the first symptoms appear, patients sense that something is wrong and begin to search for answers. In the case of RD, the absence of clinical justification and the impossibility of expressing their discomfort increases their confusion and deteriorates relationships with both family members and healthcare providers. The struggle frequently begins in childhood, when differences emerge socially and physically.

*“I knew something was not right. I couldn’t act like the other kids, and it frustrated me a lot. My mother expected the same from me as my sister, but I couldn’t reach her level”* (P2, 29 years old, cystic fibrosis)

In some cases, repeated invalidation leads to withdrawal and self‐silencing.

*“Every time I went to my doctor’s appointment, I came away deeply upset. I tried to explain as best as I could how I was feeling and what was happening to me. I always received the same response—everything was normal, there was no abnormality. I stopped going to the appointments out of shame and feeling like a liar”* (P3, 35 years old, mitochondrial disease)

Sometimes, the lack of justification leads some patients to internalize their discomfort and attribute their situation to a state of normalcy. The individual tries to adapt their limitations to daily life and conceals their illness in an attempt to be understood and approved by their immediate surroundings. Still, the longing to understand their condition remains, and each new test revives the hope of finally identifying what is behind their discomfort, even when options are running out.

*“In the end, they make you believe that your situation is normal. You don’t know how to explain what is happening to you, and you feel misunderstood by your surroundings”* (P1, 41 years old, glycogenosis)

*“All possibilities had already been exhausted. There was no way to find what was causing my limitations. I felt so much anger at having to get used to continuing with my life in this way. But every time they offered me a new opportunity to study my case, it excited me a lot”* (P6, 28 years old, Wolfram syndrome)

Receiving a diagnosis becomes a turning point in the trajectory of the patient with RD. What is usually perceived as bad news is, for them, a moment of liberation that allows directing care toward a concrete goal while reducing misunderstanding and fostering connection with others in the same situation.

*“That day was unforgettable; I remember every detail of what happened. I felt liberated; I had achieved it. All my suffering and struggle had borne fruit”* (P5, 50 years old, Ehlers–Danlos syndrome)

*“I achieved what I had been dreaming of for a long time. It was the turning point for everything to change its course. From that moment on, everything was focused on my condition; I have been able to meet people with the same disease, and I haven’t had to go through as many detours as at the beginning. It was a before and after in my process”* (P1, 41 years old, glycogenosis)

Across these quotes, searching for answers is not only diagnostic; it is a search for validation, belonging, and identity.

#### 3.1.2. Subtheme 1.2. A Complex Reality Beyond the Diagnostic Barrier

Although receiving a diagnosis helps patients frame their condition, it is only the beginning of a new struggle. The scarcity of cases and information forces them to search tirelessly for reliable sources to better understand the disease and face its limitations.

*“I thought that when I found the name of my disease, everything would be simpler. However, problems kept coming one after another, and you didn’t really know who to turn to”* (P8, 34 years old, peripartum cardiomyopathy)

*“Each time, I received different information. I had the feeling that no one cared about my disease simply because it was not well-known. Then, you search for information on your own, but you don’t know if it’s true or not. You are not sure how to proceed”* (P3, 35 years old, mitochondrial disease)

Public awareness of RD remains limited, hindering the social and occupational inclusion of affected individuals and contributing to inequitable treatment and reduced health‐related quality of life. A large proportion of RD entail functional impairment that interferes with activities of daily living, thereby requiring individualized accommodations to lessen their burden on patients and families. Nevertheless, many patients report persistent barriers to resources routinely available to the general population and restricted participation in conventional work settings.

*“I feel like a strange and misunderstood person with a disease, a disease that most of the population has, but the difference is that mine is little known. They don’t know what it is, and they are already assigning me disability”* (P7, 40 years old, complex regional pain syndrome)

*“As my disease progressed, it became increasingly difficult for me to perform my job in the same way as before. I didn’t have the necessary adaptations, and carrying out my daily tasks was a significant effort”* (P2, 29 years old, cystic fibrosis)

RD frequently disrupt educational trajectories and generate significant economic burdens, often leading to dependency on family support. These challenges, compounded by a lack of systemic resources, contribute to emotional strain and a perceived limitation of personal aspirations.

*“The moment you find out you have a rare disease, and especially in my case, which is quite debilitating, you know that you won’t be able to achieve the goals you would have set without the disease. I won’t be able to academically reach where I would have liked; it’s complicated”* (P6, 28 years old, Wolfram syndrome)

*“Having a disease like this entails a significant economic expense. If it weren’t for my parents, it would be very difficult for me to keep up with my therapy. Moreover, there comes a point where you become too dependent and need the support of another person. I feel sorry that this puts a strain on others”* (P3, 35 years old, mitochondrial disease)

These quotes highlight that diagnosis is not resolution, but reorientation, shifting the weight of uncertainty onto management, adaptation, and social visibility.

### 3.2. Theme 2. Management and Handling of RD by the Healthcare System

The healthcare management of RD is a major challenge for the healthcare community given the complexity of handling these pathologies and the scarce knowledge existing in relation to them. Moreover, the lack of clinical experience among healthcare professionals in this field has implications for the quality of care, patient follow‐up, and the treatment process. The involvement of the affected individual and their family in decision‐making is a fundamental strategy in daily therapy, serving as the guiding thread of the program imposed by the specialist involved in the case. On the other hand, difficulties in coordination between primary care services and specialized centers, as well as the lack of updates on disease‐related issues, create a gap in the patient’s recovery process. Thus, the search for new strategies to address these shortcomings is one of the priorities in the RD population.

#### 3.2.1. Subtheme 2.1. Practicing Care Under Uncertainty

RD are inherently challenging to diagnose due to their low prevalence, limited diagnostic protocols, and scarce scientific evidence. The lack of specialized experts and reference centers extends the waiting time for patients to receive a specific diagnosis. This lack of coordinated expertise frequently leads to trial‐and‐error therapeutic approaches, which not only fail to meet patients’ needs but may also intensify their sense of vulnerability, distrust, and therapeutic fatigue.

*“I visited all the hospitals in search of an answer. Each time, I encountered a different professional, and none of them provided what I needed. I felt like a puppet with no one looking out for me, being used to experiment with whatever they prescribed”* (P9, 42 years old, congenital multiple arthrogryposis)

*“I can understand that, for professionals, facing a new and unclear situation must be challenging. However, it is essential to reach an agreement to delve into these diseases and not further harm the patient”* (P4, 32 years old, eosinophilic esophagitis)

The treatment options available to healthcare professionals are limited, lacking specificity for the pathology and, in most cases, focusing on palliating symptoms. Moreover, these treatments are often expensive and have low coverage by the healthcare system, making access difficult for patients. Therefore, even if there is therapeutic hope to combat the disease, the monitoring and adherence to the action plan weaken. It is crucial for both patients and healthcare professionals to have programs and public policies that facilitate access to affordable treatment, promoting proper disease management and improving therapeutic adherence.

*“I started trying different medications, and eventually, we found one that worked well for me. However, it was impossible to sustain the treatment due to its high cost, and on top of that, I had to buy it very frequently”* (P3, 35 years old, mitochondrial disease)

*“The difficulty in accessing the necessary treatment not only adversely affects the disease but also hampers the work of healthcare professionals. We should form a collective effort with them and advocate for the implementation of programs to change the current situation”* (P5, 50 years old, Ehlers–Danlos syndrome)

Despite only a small proportion of RD having a specific and effective treatment, research directed toward this group is scarce and insufficient. As mentioned earlier, the infrequency of these conditions in society and the lack of support from various stakeholders in the process hinder research efforts. Therefore, it is crucial to invest in research and strengthen the collaboration between healthcare professionals, researchers, and patients themselves in the quest for strategies that improve the quality of life for those affected.

*“We are not of interest to society or pharmaceutical industries. Research is paralyzed due to the lack of support and funding they provide. It seems unfair not to fight for improving our day-to-day lives simply because we are a minority”* (P7, 40 years old, complex regional pain syndrome)

*“It is frustrating to think that there are active ingredients that have already been researched, with all the effort that entails, and they do not progress due to lack of support. Strategies need to be created to promote awareness of these projects in other countries and to encourage them to take action in developing these initiatives”* (P4, 32 years old, eosinophilic esophagitis)

Thus, systemic limitations intensify vulnerability and reinforce the perception of being medically expendable.

#### 3.2.2. Subtheme 2.2. Patients as Active Knowledge Holders

In the context of uncertainty, inexperience, and a lack of evaluative tools, patients with RD become an essential focus in their monitoring and the development of a personalized therapeutic plan by healthcare professionals. In the case of more debilitating conditions, caregivers take on a crucial role in redirecting treatment, providing crucial information for monitoring, and offering necessary clues to assist healthcare professionals in their work. Therefore, it is of vital importance to involve the patient and their environment in decision‐making, allowing them access to information and resources available related to the disease.

*“We are the primary source of information for healthcare professionals. We propose alternatives, accept and reject propositions, always with their consent and verification”* (P3, 35 years old, mitochondrial disease)

*“I suppose that all diseases must revolve around the patient. But I think our role in this type of disease is more relevant because there is a greater lack of knowledge, healthcare professionals are not as specialized in the treatments and do not know how we are going to react to the therapy. They are the ones suggesting that we propose options to try, and then they ask us to let them know how it went”* (P1, 41 years old, glycogenosis)

Patient and caregiver experiences represent a valuable source of practical knowledge that can inform and enhance clinical decision‐making in RD. Incorporating patient input fosters a bidirectional relationship in which healthcare professionals gain context‐specific insights, and patients assume an active role in managing their condition. This collaborative approach improves the efficiency of care, avoids ineffective interventions, and strengthens therapeutic adherence.

*“My doctor gives me a very active role in the evaluation and treatment of my illness, he always lets me tell him about my experience with therapies I have started to use or foods I have included in my routine, and he takes notes himself to share with other patients”* (P8, 34 years old, peripartum cardiomyopathy)

*“I heard about a diet that might be beneficial for my condition, and I mentioned it to the healthcare team overseeing my case. They were already familiar with it and advised against it because they knew of patients in my situation who didn′t have a positive experience with it. I left the appointment quite satisfied because, in reality, I saved myself unnecessary effort, and I can use that time to explore and try other alternatives”* (P6, 28 years old, Wolfram syndrome)

Systematic documentation of disease progression by patients plays a key role in expanding clinical knowledge and guiding research in RD. Recording symptoms, treatment responses, and personal experiences not only contributes to individualized care but also generates data essential for the design of new studies and therapeutic strategies. This patient‐derived information serves as a foundation for collaborative research efforts and the development of interventions aimed at improving outcomes for the broader RD community.

*“A research group contacted me that had a study related to my disease. They treated me very well; I had to provide them with all the information about my process, and they subjected me to a multitude of tests”* (P2, 29 years old, cystic fibrosis)

*“They have always emphasized the importance of meticulously documenting the progression of the disease, relapses, peaks of improvement, and every aspect of the treatment process. This information is crucial for continuing to advance in the understanding of the disease and for research purposes”* (P4, 32 years old, eosinophilic esophagitis)

These examples demonstrate a shift from passive patienthood to negotiated agency, where patients’ lived expertise compensates for clinical gaps.

#### 3.2.3. Subtheme 2.3. Extending Care Through Relational and Digital Spaces

The need for greater understanding and the sharing of experiences with those who know the disease process first‐hand has led patients with RD to seek new strategies to cope with their process and to make their daily lives more manageable. To cope with systemic and daily burdens, many patients turn to peer communities, social media, and telecare, expanding care beyond traditional clinical boundaries. Patient societies have become a meeting point where individuals facing a common goal connect, offer advice and useful information, and collaborate collectively to increase visibility. Healthcare professionals have become key advocates of this strategy, actively participating in the organization and development of support groups. In fact, patients highlight the usefulness of specialists being part of these meetings as part of their work schedule to gain different perspectives and provide scientific insights to those in need.

*“It’s very satisfying to hear from people who are going through a situation similar to mine and share concerns and worries that resonate with me. I look forward to attending the society every month and chatting with my fellow members”* (P1, 41 years old, glycogenosis)

*“It’s a way for professionals to firsthand immerse themselves in the daily lives of their patients, and for all of us to benefit from each other′s information and experiences. Undoubtedly, it is one of the best therapies that can be offered to us”* (P6, 28 years old, Wolfram syndrome)

The use of social media as a tool for health communication is increasingly common among both patients and healthcare professionals. For individuals with RD, these platforms offer accessible means to share experiences, disseminate updated information, and foster peer support. Patients often create dedicated channels to connect with others in similar situations, while professionals contribute by publishing educational content and offering informal guidance. This dynamic facilitates rapid knowledge exchange, strengthens social networks, and mitigates feelings of isolation.

*“Social media has been a tremendous help in coping with the illness. You see how people share their day-to-day lives, and you feel accompanied. Thanks to them, I have been able to fill the void of loneliness and misunderstanding that I felt during parts of my therapeutic process”* (P10, 41 years old, idiopathic intracranial hypertension and Tarlov cysts)

*“I have been able to contact many professionals and researchers specialized in the area of my disease. I didn′t need to schedule appointments with my doctor every time I had a question; through social media, I received a quick response. Additionally, this has helped me take the step of sharing my situation to also be of help to others”* (P6, 28 years old, Wolfram syndrome)

Telecare has become an increasingly valuable resource in optimizing healthcare delivery, particularly by reducing unnecessary in‐person visits and alleviating pressure on the healthcare system. For individuals with RD, whose follow‐up often requires frequent and burdensome appointments, remote consultations offer a practical alternative. Teleassistance enables timely, personalized care within the patient’s home environment, enhancing accessibility and continuity while minimizing physical and logistical strain.

*“Sometimes, I didn’t see the need to visit the clinic so frequently, especially when it was just to discuss my progress. In these situations, teleassistance has been very helpful for its convenience and ease”* (P3, 35 years old, mitochondrial disease)

*“At first, I was opposed to having my consultation remotely because I thought it would be more impersonal and cumbersome. However, I was pleasantly surprised by how easily my issue was resolved without the need to leave my home”* (P7, 40 years old, complex regional pain syndrome)

Together, these practices reflect a redefinition of care—less institutional, more relational and distributed.

## 4. Discussion

The aim of this study was to explore the lived experiences and perceptions of patients with RD in relation to the disease process and its management by the healthcare system. The phenomenological approach used in this study has allowed us to understand the phenomenon from personal and social perspectives. The findings revealed two overarching interpretive themes: (1) RD: a desperate struggle against abstraction and hindrance, which reflects the emotional burden, diagnostic uncertainty, and social consequences experienced by patients and (2) management and handling of RD by the healthcare system, which captures the structural, relational, and organizational barriers encountered throughout the care process.

These findings converge with cross‐disease research showing that RD populations commonly experience both diagnostic invisibility and systemic discontinuity. However, our study expands this view by illustrating how these processes are lived phenomenologically, shaping identity, belonging, and relational trust. In relation to the difficulties most frequently experienced by the RD population, establishing an accurate diagnosis represents one of the most desperate and agonizing goals for the patient. This delay in naming the disease can take several years, resulting in inadequate treatment or missed treatment opportunities, and is associated with increased morbidity and mortality [14]. In a survey conducted with relatives of patients with RD from different countries, they consulted more than five specialists and received between two and three incorrect diagnoses before receiving a correct one, extending over a period exceeding 5 years [46]. Similarly, in the study conducted on pediatric patients and their caregivers, the lengthy diagnostic odyssey included an extensive battery of tests performed on the patient, many of which were invasive and caused significant trauma to the affected individual and their relatives [32]. The complexity of attributing a diagnosis to RD has traditionally been linked to the heterogeneity and low frequency of these conditions, multisystemic involvement, and significant variability in clinical manifestations [47]. In recent years, numerous advances have been made in the field of molecular biology and genetics to achieve earlier and more accurate diagnoses, thereby improving the prognosis and outcomes for patients [48–51].

The assignment of a specific diagnosis that responds to the patient’s manifestations is the starting point of a complex path. Individuals in this group express a sense of isolation and stigma, even from some family members who may not accept the situation they have to live with [31]. According to the study by Thomson and colleagues [52], family members often chose not to believe their loved one’s symptoms because it can be extremely vulnerable to witness another person’s suffering, especially when it is invisible and more difficult to understand. On the other hand, the lack of accessibility, misunderstanding by public institution workers, and obstacles related to education and employment are some of the structural limitations associated with RD. Consistent with the results of our study, an inductive qualitative analysis of patients with RD revealed that participants feared losing their jobs, felt anger at having to distance themselves from their friends due to symptoms, and experienced despair at not enjoying equal educational opportunities [53]. Similarly, this loneliness is also evident in the scarcity of information and research on RD, leading affected individuals to use multiple resources to seek reliable information about their condition [54].

In addition to the impact of having an RD on the patient and their immediate environment, the management and approach to this group of pathologies implies a real challenge for the healthcare system. Clinical professionals have limited information and few scientific studies to confidently address RD, impacting the treatment and monitoring of patients [55, 56]. Thus, in the study conducted by Witt and collaborators (2023), patients with RD expressed how misinformation and the lack of updates from some healthcare professionals about their condition prolonged the recovery process, unnecessary treatments, and hospital stays. On the other hand, the scarce resources available and the limited collaboration with universities, the pharmaceutical industry, and government agencies further complicate the approach to RD. All of this affects drug discovery, access to treatments, and public awareness of RD [57]. In a survey of primary care and specialized doctors, the main difficulties in caring for patients with RD were identified as the lack of available treatments and the difficulty in accessing new drugs or therapies available only in some countries [55]. Research and development of orphan drugs for RD pose many challenging questions, compounded by the fact that there is currently no clear mandate to decide between ethical considerations and economic reasoning [58].

Another significant challenge for the healthcare system is responding to patients’ demands for a designated reference professional who can ensure continuity of care and for the provision of consistent and coordinated information across all healthcare providers. According to the World Health Organization (WHO), the need for interprofessional collaboration is growing, so healthcare systems should introduce policies including interprofessional education and collaborative practice in order to maximize the strengths and skills of health workers [59]. Considering that these patients require long‐term care, it is even more crucial to involve their families in this process, providing them with the training and information typically held by healthcare professionals [60].

To address the fragmentation and barriers described, both patients and healthcare systems have resorted to alternative tools that foster collaboration, coordination, and empowerment. Cross‐disease research has described registries and peer networks primarily as informational resources. Our results show they additionally function as identity‐rebuilding spaces, reducing shame and enabling therapeutic belonging. Patient registries have become key instruments to promote learning networks and research collaborations among professionals, industry, academic institutions, and patient organizations [61]. Likewise, the use of electronic resources constitutes a tool to enhance the understanding of RD as a national and international online registry system and a sharing platform for research data and findings consolidation [62, 63]. In line with the results of our research, peer support groups, whether patient‐led or facilitated by professionals, have proven useful in solving practical problems and offering emotional support [62, 64, 65]. Social media has also been used to share health information and articles of interest from various online sources, empowering patients through education and fostering discussion and shared decision‐making with their healthcare providers [66–68]. Parents of patients with RD have described social media as a source of information that healthcare professionals could not provide, as a window into the experiences of other families going through the same situation, and as an accessible source of social support [69].

Taken together, this study provides an original contribution by offering a phenomenological, cross‐disease interpretation of RD experiences. Unlike most RD studies, which focus on single‐condition cohorts, this work integrates multiple diagnoses to reveal common existential and relational patterns that transcend pathology‐specific features, highlighting implications for system‐level nursing practice and patient‐centered coordination.

### 4.1. Limitations

This study has several limitations that should be considered when interpreting the results. Regarding sample selection, the use of purposive sampling, while appropriate for phenomenological inquiry, may limit the transferability of the findings, as participants were recruited through a patient association and a clinical unit, which may not fully represent the diversity of experiences among patients with RD in other contexts. With respect to data collection, it relied solely on semistructured individual interviews; although this method offered in‐depth personal accounts, the absence of triangulation with other sources, such as focus groups, may have limited the depth and contextual richness of the interpretation. Finally, while hermeneutic phenomenology acknowledges and incorporates the researchers’ pre‐understandings, the dual role of some researchers as healthcare professionals may have influenced data interpretation, even with reflexivity practices in place. These limitations should be considered when assessing the scope and transferability of the findings.

### 4.2. Implications for Nursing Management

Nursing professionals play a vital role in supporting patients and their families throughout the diagnostic journey, treatment, and long‐term handling of RD. In the Spanish healthcare system, where this study was conducted, patients with RD are usually followed in a shared‐care model involving both primary care teams and specialist hospital units. Their involvement is essential across all levels of care, ensuring continuity and coordination. Nurses are often the first point of contact in primary care, where early detection and patient advocacy are crucial, and they also play a key role in facilitating referrals to specialized care and navigating complex healthcare pathways [39, 40]. Based on our findings, scenario‐adapted strategies may include: scheduling systematic symptom‐monitoring check‐ins in primary care for patients reporting persistent unexplained symptoms, creating a named nursing contact to support care navigation, and using structured referral pathways to reduce diagnostic detours.

To provide effective care, nurses and healthcare teams must be aware of the unique challenges and barriers faced by patients with RD, including delayed diagnosis, lack of information, and limited access to treatment. Nurses should adopt a patient‐centered approach, acknowledging the emotional and psychological impact of RD on patients and their families, and this includes providing emotional support, education, and empowerment to promote self‐management and adherence to treatment. In practice, this may involve implementing brief emotional‐screening checklists during follow‐up visits, offering anticipatory guidance to families at key points of uncertainty (e.g., while awaiting test results), and providing curated disease‐specific information sheets or digital resources to reduce misinformation and online burden. Moreover, interprofessional collaboration is critical to overcome the current fragmentation of care due to hyperspecialization and the lack of coordination between levels [59]. Nursing professionals can act as essential bridges between services, helping to ensure that care is comprehensive, coherent, and tailored to the patient’s needs. To strengthen their role, further education and training in RD are required to provide accurate information and holistic support to patients and families [39].

To operationalize interprofessional collaboration, nurses could coordinate joint case reviews between primary and specialist teams, maintain shared care plans accessible to all professionals, and track unanswered patient questions as triggers for escalation or reassessment. By acknowledging the unique challenges faced by patients with RD, nurses can significantly improve care and outcomes. Nurses may also lead community‐based RD support groups, co‐moderate online peer spaces with patient associations, and contribute to patient registries by systematically documenting symptoms, trajectories, and therapeutic responses. Drawing on our findings, we suggest concrete nursing strategies such as (i) proactive symptom monitoring and early case flagging in primary care; (ii) assignment of a named nurse to support navigation and advocacy; (iii) structured interprofessional case reviews; and (iv) nurse‐facilitated patient education, peer‐support spaces, and registry participation.

## 5. Conclusions

At first, all the efforts and hopes of individuals with RD are directed toward deciphering their symptoms and obtaining a diagnosis that provides answers to their silent suffering. However, beyond obtaining a diagnosis, patients continue to face multiple challenges, including delayed access to treatment, fragmented healthcare pathways, limited social understanding, economic burden, and difficulties maintaining educational, occupational, and social participation. The healthcare system plays a key role in managing the RD community and serves as a reference support for patients and their families. Despite this, healthcare professionals face a complex challenge where a lack of updated information, lack of experience, and limited research collaboration hinder clinical management, adversely impacting the health of users. The role of the affected individual is essential to redirect the therapeutic process and propose strategies that can bring hope to their treatment. Based on this, efforts have been made to find complementary methods to enhance the well‐being, support, and understanding of the patient, actively participating alongside healthcare professionals. Thus, it is essential for managers and healthcare professionals involved in caring for individuals with RD in clinical settings to prioritize the acquisition of specialized knowledge and diverse strategies aimed at reducing the negative effects on both the patients and the healthcare providers. Moreover, more effective strategies for resource allocation are necessary to address or alleviate the impact of RD as a public health concern.

## Author Contributions

Conceptualization: Pedro Soriano‐Martin, Isabel Font Jiménez, and Pablo Roman; methodology: Pedro Soriano‐Martin, Pablo Roman, and Cristofer Ruiz‐González; data collection: Pedro Soriano‐Martin; formal analysis: Pedro Soriano‐Martin, Cristofer Ruiz‐González, Daniel Bertini‐Pérez, and Antonio Javier Alias‐Castillo; writing–original draft: Pedro Soriano‐Martin, Daniel Bertini‐Pérez, and Pablo Roman; writing–review and editing: all authors; supervision: Pablo Roman and Isabel Font Jiménez.

## Funding

This research received no specific grant from any funding agency in the public, commercial, or not‐for‐profit sectors.

## Disclosure

All authors approved the final manuscript.

## Conflicts of Interest

The authors declare no conflicts of interest.

## Supporting Information

Additional supporting information can be found online in the Supporting Information section.

## Supporting information


**Supporting Information** Supporting File 1: Semistructured interview guide used for data collection purposes.

## Data Availability

The data that support the findings of this study are not publicly available due to the sensitive nature of the interview data and the risk of participant identification. De‐identified data may be available from the corresponding author upon reasonable request and subject to ethical approval.
