# Supplementary material for: Lived Experiences of Patients With Rare Diseases and Healthcare System Barriers: A Phenomenological Study
Source: J Nurs Manag. 2026 Jun 29;2026:6754802. doi: 10.1155/jonm/6754802 (PMC13315831; doi:10.1155/jonm/6754802)
Supplement: Supplementary file 1 — Supporting Information Supporting File 1: Semistructured interview guide used for data collection purposes. [file JONM-2026-6754802-s001.docx]

**SUPPLEMENTARY FILE 1. SEMI-STRUCTURED INTERVIEW GUIDE**

This semi-structured interview guide was designed to support an open, dialogical process aimed at understanding participants’ lived experiences of rare disease. In line with Gadamer’s hermeneutic philosophy, questions were used flexibly and adapted during the interview to allow meanings to emerge through dialogue rather than to elicit predefined themes.

Participants were encouraged to speak freely and to guide the conversation toward aspects they considered meaningful.

**Opening**

*I would like you to talk about your experience in your own words. There are no right or wrong answers. I may ask follow-up questions to better understand what you mean, but you are free to focus on what feels most important to you.*

**1. Lived experience of illness and diagnosis**

Can you tell me about the moment when you first realised that something was not right with your health? (What do you remember most vividly from that period?)

How did you experience the time between the onset of symptoms and receiving a diagnosis? (How did you try to make sense of what was happening to you during that time?)

In what ways did this period affect your everyday life, relationships, or sense of yourself?

Can you describe your experience of receiving the diagnosis? (How did that moment change your understanding of your situation?)

**2. Experiences with the healthcare system**

How would you describe your interactions with healthcare professionals throughout your illness process?

What experiences stand out to you as particularly helpful or difficult in your contact with the healthcare system? (How did these encounters influence your trust or expectations?)

How did you experience communication and information provided by healthcare professionals about your condition?

From your perspective, how did the organisation of care affect your experience of living with a rare disease?

**3. Meaning-making, support, and relational experiences**

What role have other people played in helping you understand or cope with your illness (family members, friends, caregivers)?

Have you had contact with other people living with a similar condition? (How did sharing experiences with others affect you?)

How would you describe the emotional or social challenges you have faced, and how you have dealt with them?

**4. Use of digital tools, peer communities, and social media**

Have digital tools, online platforms, or social media been part of your experience of living with a rare disease?

What motivated you to seek information or connection through these channels?

How have these digital or online spaces influenced how you understand your illness or manage daily life?

Have you encountered any difficulties or negative experiences related to online information or interactions? (How did you navigate or interpret these situations?)

How do you relate information found online to the information provided by healthcare professionals?

**5. Reflections and future perspectives**

Looking back on your experience, what moments or aspects feel most significant to you now?

What kinds of support do you think would be most helpful for people living with rare diseases?

From your experience, what role should healthcare professionals—particularly nurses—play in supporting patients with rare diseases?

Is there anything you would like to change or improve in how care is currently provided for people with rare diseases?

**Closing question**

Is there anything else you would like to share that you feel is important for understanding your experience?

*Thank you very much for sharing your experiences.*
